# Attenuation Correction for Small Animal PET Images: A Comparison of Two Methods

**DOI:** 10.1155/2013/103476

**Published:** 2013-04-16

**Authors:** Daniela D'Ambrosio, Federico Zagni, Antonello E. Spinelli, Mario Marengo

**Affiliations:** ^1^Medical Physics Department, University Hospital S. Orsola-Malpighi, Via Massarenti 9, 40138 Bologna, Italy; ^2^Medical Physics Department, IRCCS Fondazione Maugeri, Via Salvatore Maugeri 4, 27100 Pavia, Italy; ^3^Medical Physics Department, IRCCS San Raffaele, Via Olgettina 60, 20132 Milano, Italy

## Abstract

In order to extract quantitative parameters from PET images, several physical effects such as photon attenuation, scatter, and partial volume must be taken into account. The main objectives of this work were the evaluation of photon attenuation in small animals and the implementation of two attenuation correction methods based on X-rays CT and segmentation of emission images. The accuracy of the first method with respect to the beam hardening effect was investigated by using Monte Carlo simulations. Mouse- and rat-sized phantoms were acquired in order to evaluate attenuation correction in terms of counts increment and recovery of uniform activity concentration. Both methods were applied to mice and rat images acquired with several radiotracers such as^18^F-FDG, ^11^C-acetate, ^68^Ga-chloride, and ^18^F-NaF. The accuracy of the proposed methods was evaluated in heart and tumour tissues using ^18^F-FDG images and in liver, kidney, and spinal column tissues using ^11^C-acetate, ^68^Ga-chloride, and ^18^F-NaF images, respectively. *In vivo* results from animal studies show that, except for bone scans, differences between the proposed methods were about 10% in rats and 3% in mice. In conclusion, both methods provide equivalent results; however, the segmentation-based approach has several advantages being less time consuming and simple to implement.

## 1. Introduction

Positron emission tomography is a quantitative imaging technique, capable to provide accurate values of radiotracer concentration in each voxel of the reconstructed volume. The knowledge of radiopharmaceutical concentration is important because, combined with adequate mathematical models, it allows to evaluate several physiological parameters of interest, such as perfusion, glucose metabolic rate, and receptors density [1]. In order to obtain an accurate quantification of radiotracer concentration, several physical factors must be taken into account, such as attenuation (AC) [2], scatter [3], and partial volume correction (PVC) [4]. In this work, the attention was focused on attenuation correction for small animal PET images, which is well known to be relevant for human patients [5] but could be also significant for small animals as well. Attenuation correction has been extensively discussed in the literature for human PET and SPECT studies; however, only few papers focus on AC for small animals [6–11]. 

There are several methods to obtain attenuation maps that could be employed to correct PET images for attenuation. They are mainly grouped into two categories: transmission and transmissionless methods. 

The first category of methods is based on the acquisition of a transmission image of the subject. The most used approaches are based on (1) transmissive image using, for example, rotating or annular ^68^Ga/^68^Ge sources, (2) segmented transmissive image, and (3) X-ray CT image. Transmission scanning can be performed both in coincidence and in single mode.

A quite simple approach consists of using an annular ^68^Ga/^68^Ge source surrounding the object and acquiring a transmission image in coincidence mode; however, the resulting image can be very noisy. An alternative approach is to use a rotating rod source; in this case, scatter and random coincidences decrease because only the lines of response (LORs) collinear with the rod source are accepted. The rod source is generally very active, and, thus, the detector near the source exhibits a high dead time causing an important loss of counts [13]. The same problem happens also in the case of single photon source; in order to acquire a good quality transmissive image using a fully 3D scanner, a point source with a very high radioactivity concentration must be used. This problem can be solved with use of collimated, single photon, point sources [14, 15]. However, the single mode acquisition method has also several disadvantages: (1) a significant scatter component is included in the transmissive image, and (2) an energy scaling of the attenuation map is needed because of the different photon energy of the transmissive source. For example, ^137^Cs emits 662 keV photons. In this case, attenuation maps can be obtained segmenting transmissive images, and the known attenuation coefficients at 511 keV are assigned to each of the segmented regions [16, 17]. The last and most used category of transmission methods is based on the acquisition of CT images. Analogously to the method described before, energy scaling of the attenuation coefficients to 511 keV is needed.

Transmission methods are more accurate when estimating attenuation maps because they take into account the inhomogeneities of the object attenuation coefficients. Each of them has also several drawbacks; they are more time-consuming, as the animal has to be anesthetized for a long time and also receives larger doses of radioactivity compared to transmissionless methods. Transmissive PET images do not suffer from coregistration problems that may introduce artifacts in the attenuation map, but they can be very noisy. In order to reduce the noise in the transmissive PET images, segmentation was introduced, but other problems linked to time and dose still remained.

Transmissionless methods are carried out with manual or automatic delineation of body edges directly on emissive image assuming a uniform distribution of attenuation coefficients inside the object [18, 19], or by automatic image segmentation using a fuzzy clustering algorithm [20]. Transmissionless methods are less accurate in delineating the edges and provide a less detailed attenuation map, but they have the advantages of being less time consuming, do not increase the dose to the animal, and allow for noiseless attenuation maps. Moreover, coregistration procedure is not required, and thus the attenuation map are less sensitive to image artifacts.

In this work, two attenuation correction methods are presented: a CT-based method and a transmissionless AC method based on segmentation of PET data. We firstly estimated the magnitude of attenuation effect in small animals using mice and rat phantoms. In order to implement the X-ray based method, we calibrated the small animal CT scanner and we obtained a correspondence between CT and 511 keV attenuation coefficients. In order to evaluate the accuracy of the CT-based method with respect to beam-hardening, we performed Monte Carlo simulation for rat-sized objects. As CT scan adds either acquisition time or dose to the animal, we propose a further method based on segmentation of PET images. PET data segmentation may not provide accurate attenuation maps because, depending on the radiotracer used, the body edges are not always well delineated. We acquired images using several radiotracers such as ^18^F-FDG, ^11^C-acetate, ^68^Ga-chloride, and ^18^F-NaF and evaluated the accuracy of the PET-based method with respect to CT-AC.

## 2. Methods and Materials

### 2.1. Photon Attenuation

As it is well known, transmission of 511 keV photons crossing matter is expressed by an exponential law formulated as follows:

(1) $$\begin{array}{c} \Phi =\Phi_{0}e^{-\int_{L}^{}\mu (x,y)dl}, \end{array}$$

where Φ and Φ_0_ are the transmitted and incident photon fluences, *L* is the path of the photon through the object, and *μ*(*x*, *y*) is the linear attenuation coefficient map.

The exponential term, which we refer to as *P*, is the probability that a photon reaches the detector. In PET, events are not detected if at least one of the two annihilation photons is absorbed by the object. In this case, the number of events for each LOR is reduced. The probability that an annihilation event is recorded is given by the product of the probability of each photon reaching the detectors, as expressed by the following equation:

(2) P=P1P2=e−∫L1(s,ϕ)μ(x,y)dle−∫L2(s,ϕ)μ(x,y)dl=e−∫L(s,ϕ)μ(x,y)dl,

where *L* = *L*_1_ + *L*_2_, *ϕ* is the projection angle, and *s* is the radial position. As we can see in (2), the probability depends on the total length of the path of the two annihilation photons in the object that in PET is the object thickness along the LOR. The value of *L* is related to the projection angle and the radial position. The equation describing the measured projections is the attenuated Radon transform, formulated as follows:

(3) p(s,ϕ)=∫L(s,ϕ)f(x,y)e−∫L(s,ϕ)μ(x,y)dldr,

where *p*(*s*, *ϕ*) are the projection data at angle *ϕ* and bin *s*, *f*(*x*, *y*) is a function representing the original activity concentration image, *μ*(*x*, *y*) is the attenuation coefficient map, and *L*(*s*, *ϕ*) represents the LOR.

### 2.2. Systems Descriptions

PET images were acquired with the eXplore Vista preclinical PET tomograph (General Electric) [21]. The scanner allows to acquire PET images using three different energy windows (100–700, 250–700, and 400–700 keV). For our purposes, all images were acquired using the 400–700 keV energy window. The scanner detector system consists of 36 detector blocks, arranged in two rings of 11.8 mm diameter. Each block is a 13 × 13 array of 1.45 mm square phoswich crystals: a Lutetium Yttrium Orthosilicate (LYSO) crystal of 7 mm depth optically coupled to an 8 mm depth crystal of Gadolinium Orthosilicate (GSO). This design allows to obtain depth of interaction information [22]. The transverse and axial field of view (FOV) are equal to 6.9 cm and 4.6 cm, respectively. No corrections for randoms and scatter were performed in this study. All the images were reconstructed using 2D-OSEM algorithm [23] after Fourier rebinning (FORE) [24]. CT images were acquired using the eXplore Locus small animal CT tomography (General Electric). The tube voltage was 80 kV and the current was 450 *μ*A. The system is characterized by a fixed anode with a Tungsten target source. The X-ray detector is a CCD array coupled to a Cesium Iodide (CsI) scintillation crystal. The scanner allows to acquire and to reconstruct images with different pixel binning and, thus, with different resolution levels (27–93 *μ*m). The transaxial FOV dimension is equal to 8 cm for acquisition at 93 *μ*m resolution. The axial FOV is the same for all acquisition protocols, and it is equal to 4.5 cm. For our purposes, all images were acquired using a 93 *μ*m resolution. Image reconstruction was performed with the Feldkamp cone beam algorithm [25].

### 2.3. Attenuation Correction Methods

**Figure 1:**
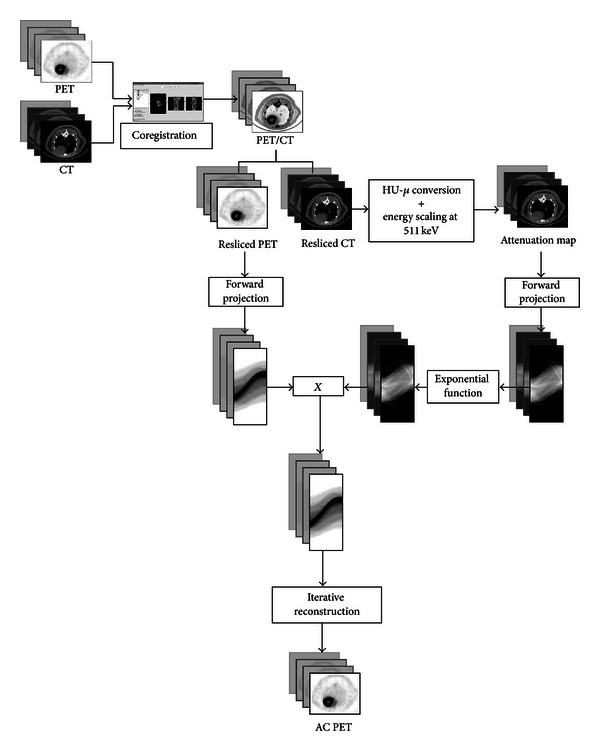
Schematic diagram of CT-AC method. CT-AC can be divided into three steps: coregistration, attenuation correction accordingly to the attenuated Radon transform (see (3)), and iterative reconstruction. More details can be found in Section 2.3.1.

**Figure 2:**
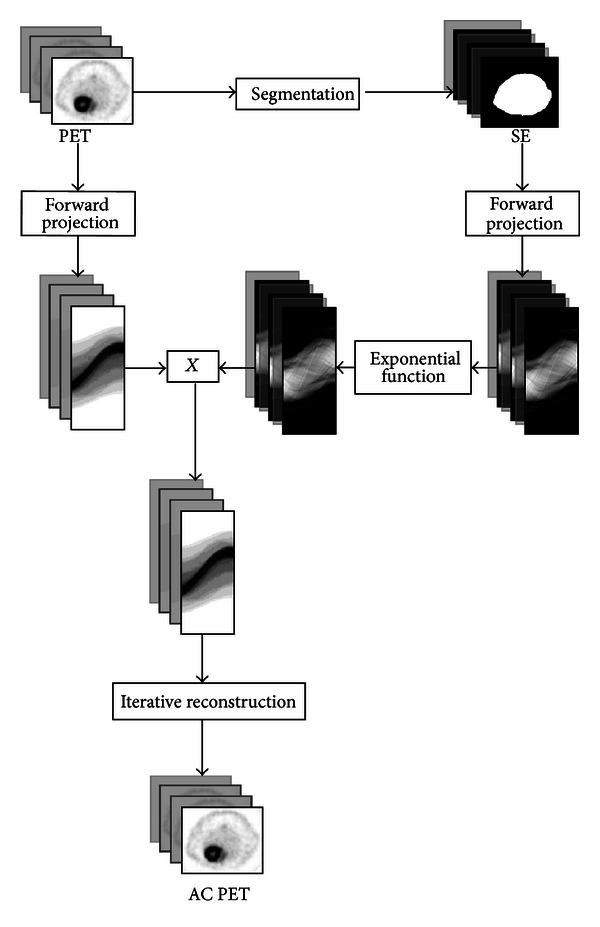
Schematic diagram of SE-AC method described in Section 2.3.2.

All the images acquired with the eXplore Vista were attenuation-corrected using the CT and SE methods as described later. The schematic diagrams of CT-AC and SE-AC methods are showed in Figures 1 and 2. 

#### 2.3.1. CT-AC Method

The CT AC method is based on a conversion between HU and the corresponding linear attenuation coefficients at 511 keV. CT images are useful because they provide detailed attenuation coefficients maps. The most important problem when using CT images is the beam hardening effect. In order to investigate the accuracy of the CT AC method, we used PeneloPET, a Monte Carlo code for PET simulations based on PENELOPE [27]. The geometry and composition of both rat-phantoms and eXplore Vista scanner were implemented to match those employed in the real acquisitions. We chose to simulate only the rat-phantom because the beam hardening effect is more important in this case than in mouse-sized objects. The CT-based attenuation corrected image was compared with images simulated without attenuation material. On both images, line profiles and region of interest (ROI) analysis were performed. The calibration procedure of the CT was performed by using the inserts of Mini CT QC Phantom 76–430 of Nuclear Associates. The inserts are cylinders having different values of density and linear attenuation coefficients at 511 keV. The data published by the National Institute of Standards and Technology are in Table 1 [26]. CT-AC was implemented using Matlab, and more precisely, PET and the calibrated attenuation map images were forward projected in order to perform attenuation correction according to the attenuated Radon transform (see (3)). 

A CT image of each cylinder was acquired and a small (ROI) was drawn on the image in order to calculate the mean value of HU. The attenuation coefficients at 511 keV were plotted with respect to the measured HU, and a quadratic fit to the data was performed.

#### 2.3.2. Segmentation of Emission Image AC Method

Segmentation of emission image by a global threshold was performed by means of a Matlab code, in order to obtain a 2-level image, corresponding to air and soft tissues. For PET images of phantoms, the contours are always clear, and, thus, the segmentation is quite easy to perform. In animal PET images, the detection of body edges depends on the biodistribution of the radiotracer injected. To assess applicability of the SE method, PET images with different radiotracers were acquired. 

### 2.4. Phantom Data

In order to evaluate the effect of photon attenuation in PET images and the accuracy of the attenuation correction methods described in previous section, several phantoms studies were acquired. Two cylindrical phantoms with different diameters were filled with different concentration of [^18^F]-fluorodeoxyglucose (^18^F-FDG) and scanned in both PET and CT modality:

- a mouse-sized phantom, filled with uniform activity concentration;
- a rat-sized phantom, filled with uniform activity concentration;
- a rat-sized phantom with a hot sphere inside (sphere to background ratio equal to 4).

A syringe of 30 mm diameter filled to 30 cc was used as mouse-sized phantom, while a cylindrical phantom with diameter of 50 mm and length of 80 mm was used as rat-sized phantom. The mouse- and rat-sized phantoms were filled with a solution of ^18^F-FDG with activity concentrations of about 1 MBq/cc and 0.5 MBq/cc, respectively. Several zeolites soaked with a small amount of ^18^F-FDG (about 5 MBq/cc) were fixed around the two phantoms in order to use them as fiducial marks in the coregistration procedure [28]. For each phantom, a 20-minute PET scan and a 6-minute CT acquisition were performed. In order to align PET and CT, the two images were coregistered using rigid body transformation implemented in AMIDE [29].

In order to measure the improvement obtained with AC, line profiles and ROI analysis were performed. The recovery values RV for each AC method were calculated with the following equations:

(4) $$\begin{array}{c} \text{R}{\text{V}}_{\text{CT}}=\frac{\text{RO}{\text{I}}_{\text{A}{\text{C-CT}}_{}}-\text{RO}{\text{I}}_{\text{NC}}}{\text{RO}{\text{I}}_{\text{A}{\text{C-CT}}_{}}},\\ \text{R}{\text{V}}_{\text{SE}}=\frac{\text{RO}{\text{I}}_{\text{A}{\text{C-SE}}_{}}-\text{RO}{\text{I}}_{\text{NC}}}{\text{RO}{\text{I}}_{\text{A}{\text{C-CT}}_{}}}, \end{array}$$

where ROI_NC_, ROI_AC-CT_, and ROI_AC-SE_ are the mean values in a ROI drawn on the uncorrected image, on the AC-CT, and on the AC-SE image, respectively. In order to evaluate the shape recovery, we calculated also the flatness of the profile (i.e., the relative difference in the number of counts between the edges and the centre of an uniform phantom) with the following formula:

(5) $$\begin{array}{c} F=\frac{C_{\max}-C_{\min}}{C_{\min}}, \end{array}$$

where *C*_max⁡_ is the maximum and *C*_min⁡_ is the minimum of the count profile. The rat-sized phantom with a hot sphere was used to simulate a PET image of a hot lesion. The recovery values RV in the sphere for each AC method were evaluated.

### 2.5. Animal Data

In order to test the attenuation correction methods and particularly the SE-AC on animal images, PET and CT scans of mice and rats were performed using different radiotracers, being all animals studies approved by the Ethical Committee of our institution:

- 1 rat and 2 mice were injected with ^18^F-FDG (an oncological and a cardiac study);
- 3 further rats were injected with ^18^F-NaF, ^11^C-acetate, and ^68^Ga-chloride.

All animals were anesthetized with 3%–5% of sevoflurane and 1 l/min of oxygen and were injected intravenously in the tail with activity of about 30 and 50 MBq, respectively. PET acquisitions of 20 minutes were performed after an uptake time depending on the radiotracer. The uptake was of about one hour for ^18^F-FDG and ^18^F-NaF studies, and about 30 minutes for ^68^Ga-chloride acquisitions. The ^11^C-acetate images were acquired immediately after the injection. A CT scan of 6 minutes was then performed for each animal after PET acquisition. PET and CT images were coregistered in order to perform AC. The segmentation process was applied on each PET image, and the SE-AC method was tested on animals studied with different radiopharmaceuticals. In order to compare the two methods, CT-AC was performed for each image as well. We analyzed all the images performing profiles across an axial slice of a particular anatomical region related to the radiotracer used and calculating the mean value in the anatomical ROI. Thus, we calculated the recovery values according to (4) and evaluated the segmentation-based AC method on heart, spinal column, liver, and kidneys using ^18^F-FDG, ^18^F-NaF, ^11^C-acetate, and ^68^Ga-chloride, respectively.

## 3. Results

### 3.1. CT Images Calibration

**Figure 3:**
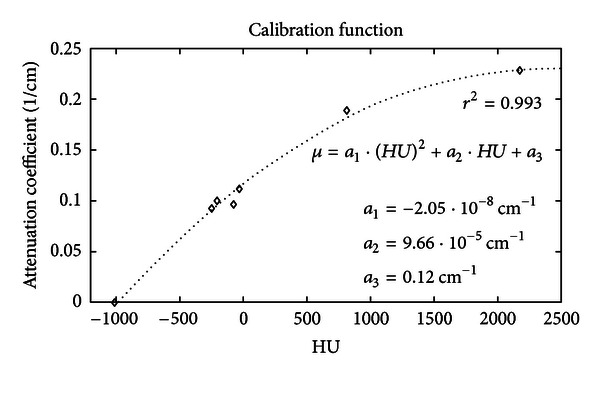
Calibration function to convert HU into attenuation coefficient at 511 keV. Attenuation coefficient at 511 keV is plotted with respect to CT numbers. This function is used to create the attenuation map to correct PET images.

In this section the results of CT images calibration are presented. The calibration function (Figure 3) is given by the following equation:

(6) $$\begin{array}{c} \mu =a_{1}{\left(\text{HU}\right)}^{2}+a_{2}\text{HU}+a_{3}, \end{array}$$

where *μ* is the attenuation coefficient at 511 keV, HU is the CT number measured from the image, and the fit coefficients are *a*_1_ = −2.05 · 10^−8^ cm^−1^, *a*_2_ = 9.66 · 10^−5^ cm^−1^, and *a*_3_ = 0.12 cm^−1^. The correlation coefficient *r* ^2^ was equal to 0.993.

### 3.2. Evaluation of CT-Based Attenuation Map by Monte Carlo Simulation

**Figure 4:**
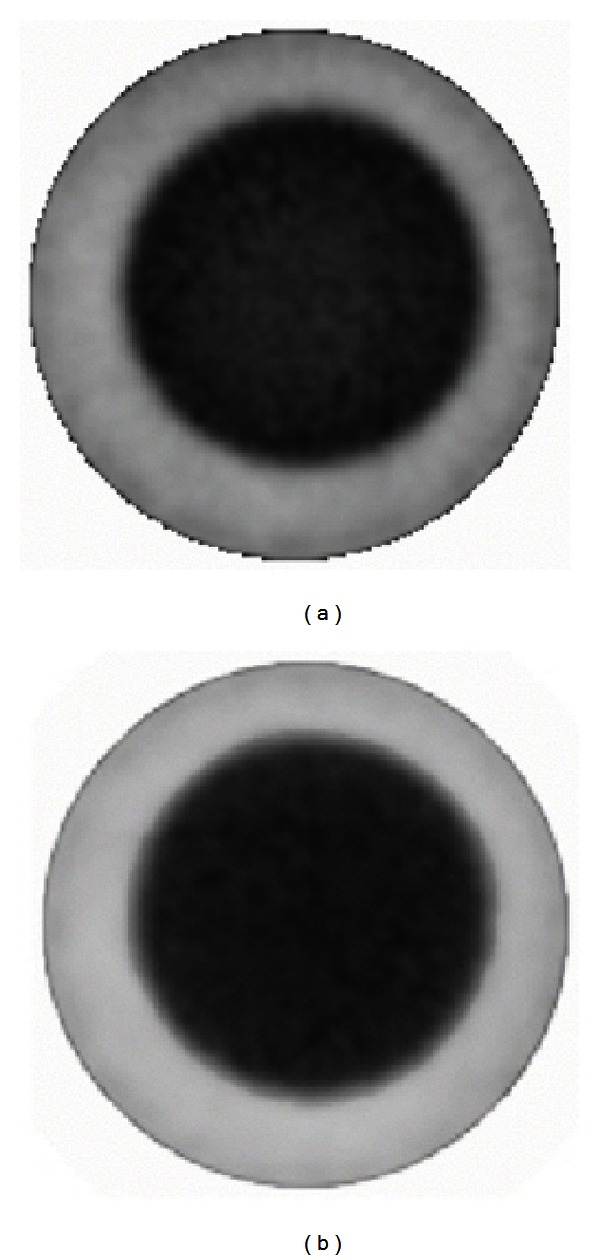
Transaxial slices of rat-phantom real PET images: original image (a) and attenuation corrected image using CT-based AC method (b). Note the uniformity in the AC image.

**Figure 5:**
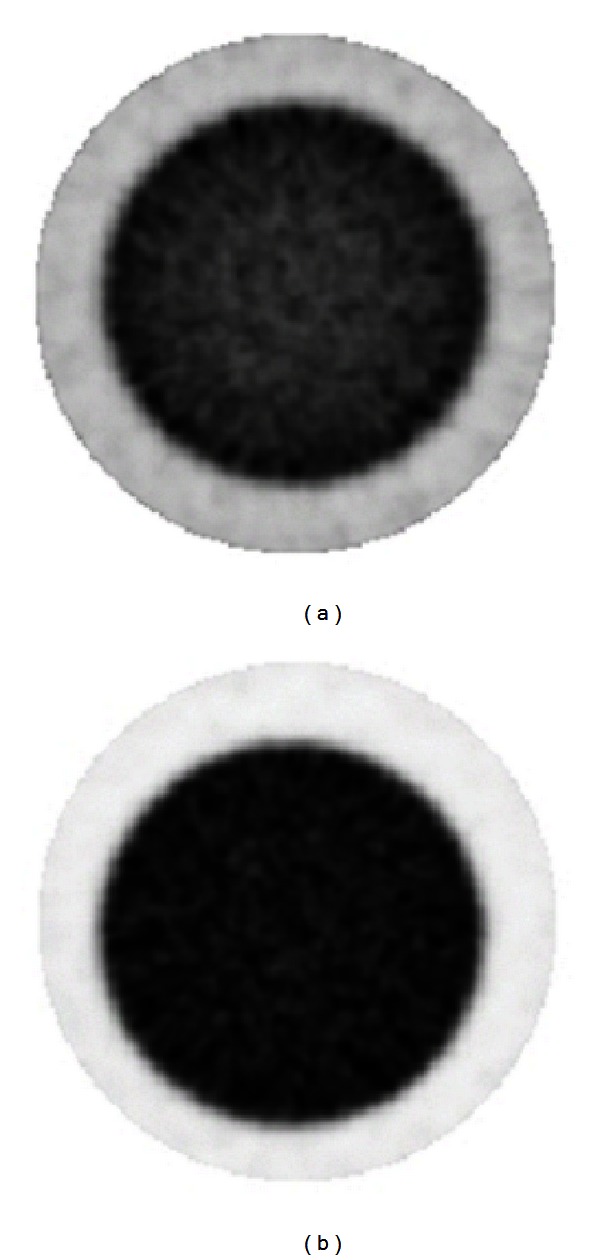
Transaxial slices of rat-phantom simulated PET images using PeneloPET. On the left side the image resulting from the simulation of uniform activity concentration with attenuation (a); on the right side the simulated image of uniform activity concentration without attenuation (b). Only in this case images were reconstructed using OSEM 3D [12].

**Figure 6:**
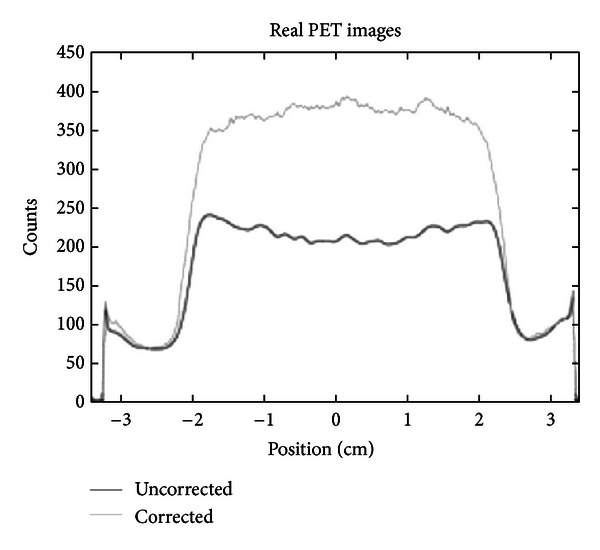
Profiles traced across the center of a transaxial slice of images reconstructed from PET acquisitions of a real rat-phantom.

**Figure 7:**
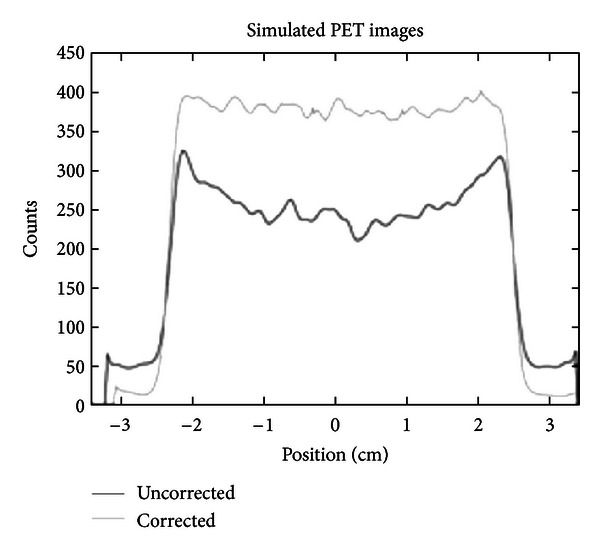
Profiles traced across the center of a transaxial slice of images reconstructed from PET acquisitions of a simulated rat-phantom.

In this section, axial slices of real and simulated PET images, before and after CT-AC correction, are shown. More precisely, Figure 4 shows a transaxial slice of original PET and CT-AC images of the rat-sized phantom. Profiles traced across them are shown in Figure 6. They show a count increment of about 40%, and the flatnesses calculated according to (5) for corrected and uncorrected images are about 1% and 22%, respectively.  Figure 5 shows an axial slice of simulated PET images with and without attenuation. Profiles traced across the simulated images (see Figure 7) show an increment of counts and a shape recovery. In this case, the flatness of the profile across the uncorrected image is equal to 23%, and the counts increment without attenuation is also of the order of 40%. Then, the simulations predict the same behaviour with regard to attenuation as seen with the CT-AC procedure, thus validating the CT-AC method.

### 3.3. Phantom Studies

**Figure 8:**
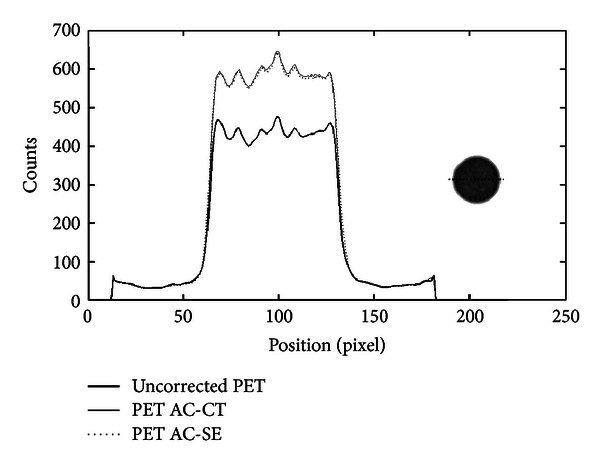
Profiles traced across the center of a cylindrical mouse-sized phantom filled with ^18^F-FDG. The number of coincidences is plotted with respect to the position across the field of view. The thick line shows the profile across the original PET image, the dotted line shows the profile across the SE-AC image, and the full line is the profile across the image corrected with the CT-AC method.

**Figure 9:**
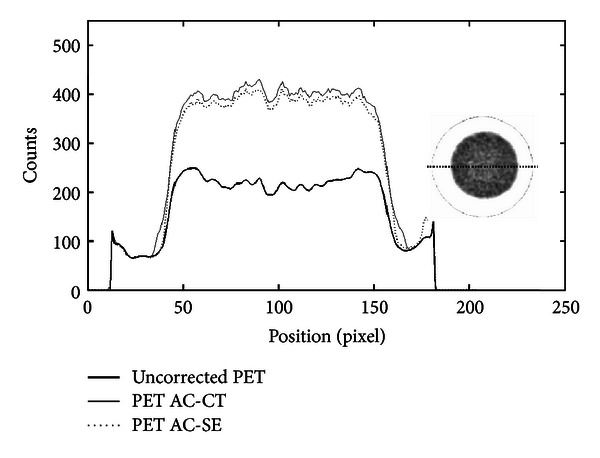
Profiles along the center of a rat-sized phantom uniformly filled with ^18^F-FDG. The plot shows three lines; thick line is the count profile of the uncorrected PET image, and the dotted and full lines, respectively, show the profiles of the PET images corrected with the SE-AC and CT-AC method. A significant increment of counts and shape recovery for both AC images is clearly seen.

Figures 8 and 9 show profiles traced across the center of a transaxial slice of pre- and postcorrected images of the mouse-sized phantom filled with uniform activity concentration. As one can see, there is a significant improvement of image uniformity after AC. In particular, the profiles show the increment of coincidences and shape recovery.  Table 2 summarized the counts measured in a ROI at the center of the slice and the flatness of the profile for the original PET, CT-AC, and SE-AC images for both phantom studies.

**Figure 10:**
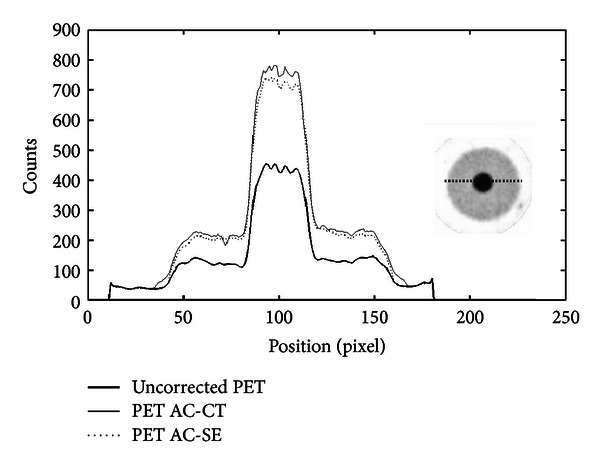
Counts measured on a line across the hot sphere using a cylindrical rat sized phantom with a hot background (sphere to background ratio is approximatively equal to 4) against position in the field of view.

The values of the RV obtained from (4) show for both mouse- and rat-sized phantoms a significant count recovery. The differences between CT-based and SE-based AC RV are less than 2% and 3% for mouse and rat phantoms, respectively. Profiles traced across the images and flatness values show an improvement between pre- and postcorrected images. In rat-sized phantom, we observe the typical cupping artifact. After AC, the cupping disappears and count recovery is evident. As explained in the previous section, acquisition of a rat phantom including a hot sphere was performed.  Figure 10 shows profiles across the hot sphere. The recovery of counts (calculated using (4)) in the hot sphere with respect to the original image for CT-AC, and SE-AC methods is showed in Table 2. The difference between the two methods is about 7%.

### 3.4. Animal Studies

**Figure 11:**
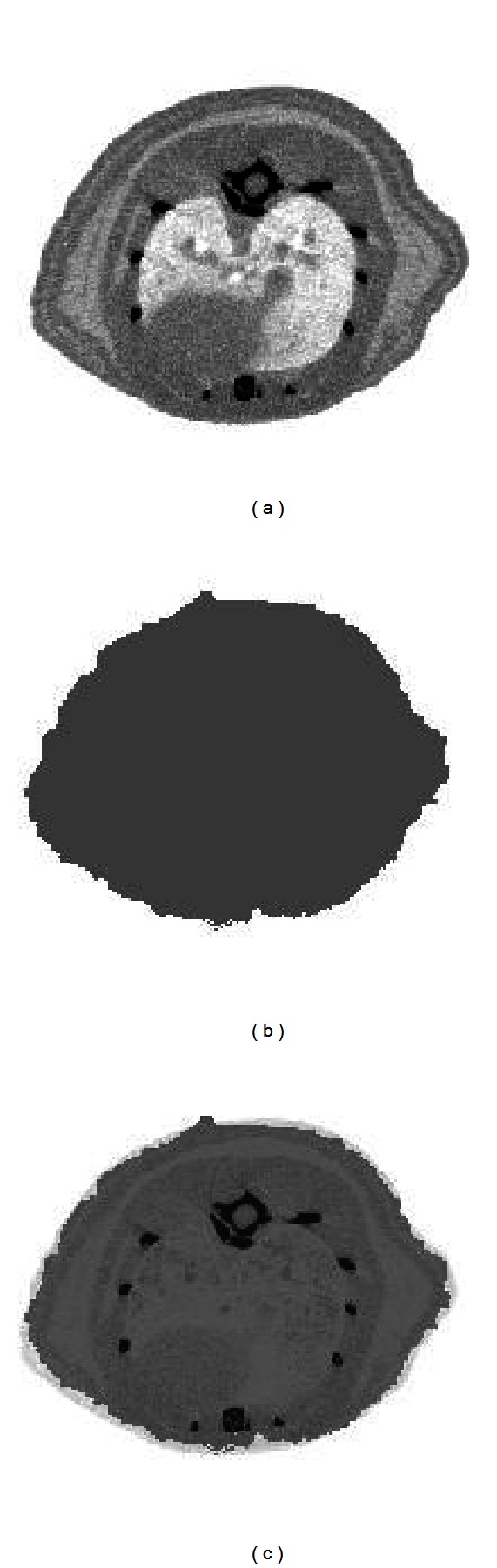
Transaxial slices of rat images: CT image (a) and segmented PET image (b). The overlay of the two previous images (c) shows how much the segmented PET image differs from the CT image.

In this section, results of attenuation correction of small animal images injected with several radiotracers are presented.  Figure 11 shows an example of CT image, segmented PET image, and the overlay between them. 

On each graph, an axial slice of the animal image is displayed showing the position of the line profile. Moreover, for each animal study, a table summarizing the average values of specific ROI counts of uncorrected and corrected images is shown. The RV calculated from (4) is reported in each table. The thick line is traced across the original uncorrected image, and the dotted and full lines were traced across the SE-based and CT-based AC images, respectively. The position of the line profile in the PET image is shown in the right side of the plot.

**Figure 12:**
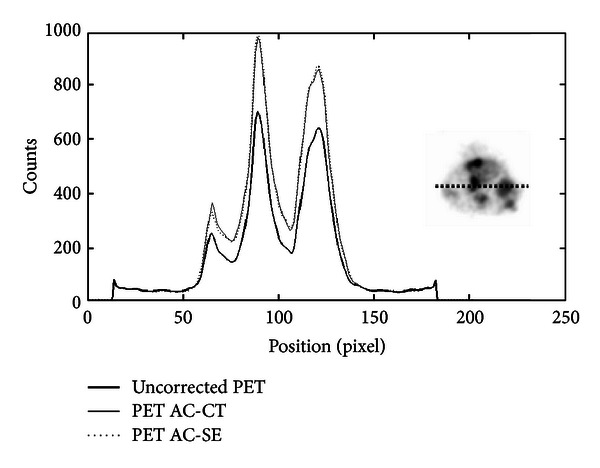
Counts profile across the mouse tumour for the uncorrected ^18^F-FDG PET image (thick line) and for the two corrected images (full and dotted lines refer to CT-AC and SE-AC images, resp.) The peak on the right side of the profile is relative to the tumour. The image on the right shows the profile position.

**Figure 13:**
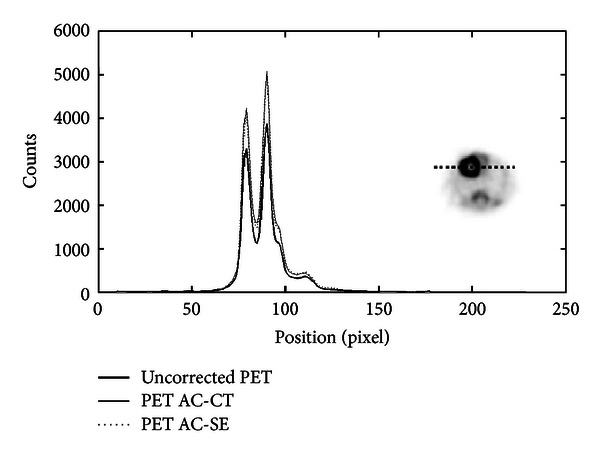
Profiles traced across mouse heart PET images acquired using ^18^F-FDG. More precisely, thick, dotted, and full lines were traced on uncorrected PET, SE-AC, and CT-AC images.

Figure 12 shows the profile across the mouse tumour. The peak on the right side of the profile is relative to the tumour. As one can see, the recovery of counts for both AC images is significant.  Table 3 shows counts measured in an ROI inside the tumour. The same analysis was performed on cardiac PET image of a mouse in order to verify the performance of SE-AC method in the quantification of tracer uptake in the myocardium and in the left ventricle.  Figure 13 shows the profile traced across the mouse heart. The two peaks in both AC images are well recovered, and, furthermore, they are very similar. More precisely, in Table 4, we show the average value of counts in the myocardium and left ventricle for the three images and the relative recovery value for the two AC methods calculated with (4). The differences between RV obtained from the two methods are less than 3%.

**Figure 14:**
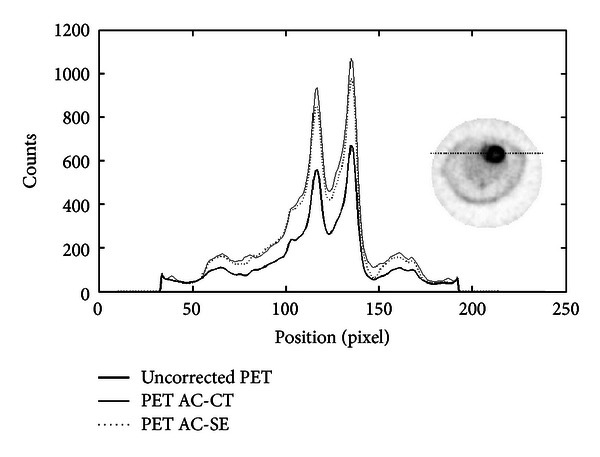
Profiles traced across the rat heart on ^18^F-FDG PET image. The position of the profile on the image is shown on the right side of the plot. The thick line represents the counts measured across the uncorrected PET image, the dotted line is for the AC method based on PET segmentation, and the full line is the profile drawn on the CT-AC image.

In order to evaluate the effect of attenuation on cardiac rat images, the same analysis on the myocardium and in the left ventricle was performed on cardiac ^18^F-FDG PET image of a rat.  Figure 14 illustrates the profiles traced across the rat heart. In Table 5, the mean value of counts on the uncorrected and corrected images and the relative RV are shown. The differences between the SE-AC and CT-AC methods are less than 9%. As discussed in Section 2.3.2, when using ^18^F-FDG, it is possible to see clearly the animal edges and, thus, to perform the segmentation of the PET image to obtain the attenuation map. 

**Figure 15:**
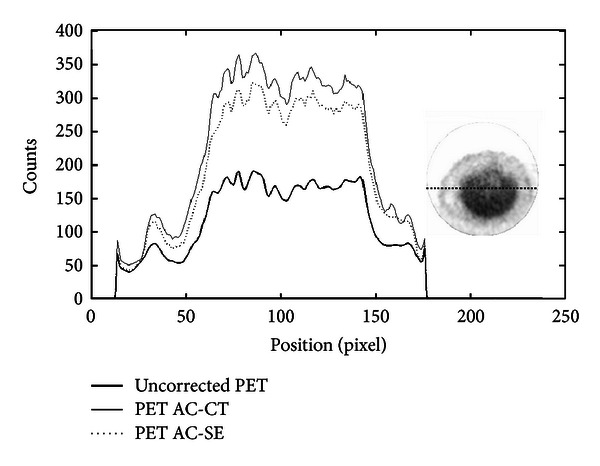
Profiles traced across the liver from ^11^C-acetate rat PET images. The thick line corresponds to the uncorrected image, while the dotted and full lines correspond to SE and CT-based AC images.

In order to investigate the use of the SE-AC method with other radiopharmaceuticals, we evaluated the performance of the AC methods also on ^11^C-acetate, ^68^Ga-chloride, and ^18^F-NaF images. For each of the these radiopharmaceuticals, a table and line profile plots are shown.  Table 6 shows the average value of counts calculated using an ROI drawn inside the rat liver on ^11^C-acetate image and the relative RV obtained with the two AC methods. The difference between the two methods is around 11%.  Figure 15 shows the profiles drawn across the rat liver on ^11^C-acetate image. The SE-AC method provides good results also for ^11^C-acetate images.

**Figure 16:**
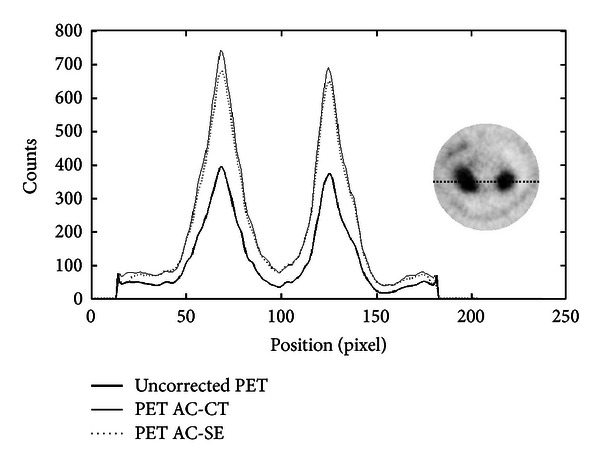
Line profiles across the kidney of the ^68^Ga-chloride rat images. The position of the line is shown on the right side of the plot. The thick line corresponds to the uncorrected PET image. The dotted and full lines correspond to the SE-AC and CT-AC images, respectively.

**Figure 17:**
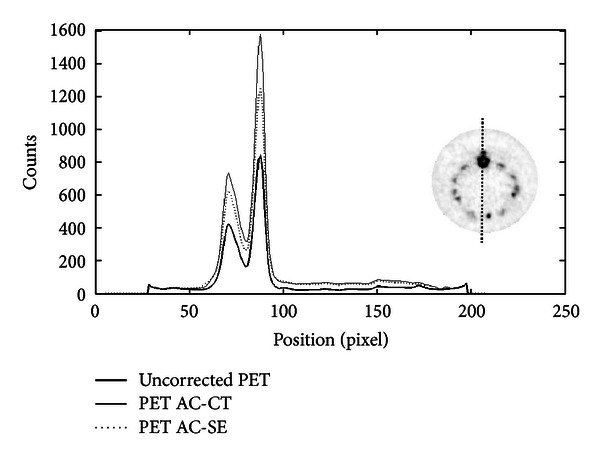
Profiles traced on ^18^F-NaF rat images across the spinal column. The position of the line is shown on the right side of the plot. The thick line corresponds to the uncorrected PET image, and dotted and full lines correspond to SE-AC and CT-AC images, respectively.

Figure 16 and Table 7 show the results of the two AC methods for a ^68^Ga-chloride image. Note that the profiles traced across the two AC images are very similar; the difference of RV between the two AC methods is approximately 7%. Segmentation of ^18^F-NaF image to create the attenuation map is more difficult to obtain. The difference between the RV calculated for both CT-AC and SE-AC methods amounts to 20%. Profiles are shown in Figure 17, and results are shown in Table 8.

## 4. Discussion

In order to achieve accurate estimates of radiotracer concentration, corrections for attenuation, scatter, and partial volume effects are necessary. Our main objectives were to assess the importance of attenuation in small animal PET images. On average, our results showed that the count recovery after attenuation correction with respect to uncorrected images is about 20% (40%) for mice (rats) images. Two AC methods were evaluated, namely, a CT-based and a segmented-emission-image-based AC. 

Similar results were also obtained by El Ali and colleagues [30]. In their work, they evaluated attenuation correction in small animals showing an underestimation of true activity concentration of about 10%–20%. They also showed that PET-based and CT-based methods provide comparable results for attenuation compensation in PET while uniform AC method may be applied only if less accuracy is acceptable. 

In our study, we acquired several phantoms, mice, and rats images. We also tested the SE-AC on images acquired using several radiotracers such as ^18^F-FDG, ^18^F-NaF, ^11^C-acetate, and ^68^Ga-chloride.

In order to implement the CT-AC method, we performed a calibration of GE eXplore Locus CT system to convert CT numbers to attenuation coefficients at 511 keV. Therefore, we were able to create attenuation maps at the correct energy. As the X-ray beam of the CT system employed is polyenergetic, the beam-hardening effect could lead to artifacts influencing the quality of the CT image and consequently the accuracy of the attenuation map. For this reason, we performed Monte Carlo simulation in order to evaluate the difference between CT-AC and MC simulations. The difference between the flatness of true CT-AC PET image and AC simulated image is about 3% showing that beam hardening effects have a negligible influence in CT-AC.

In order to test both AC methods, we acquired several phantoms. A mouse-sized phantom filled with uniform activity concentration was acquired to quantify the importance of attenuation effects and to measure the shape recovery. We acquired also a rat-sized phantom uniformly filled with ^18^F-FDG, and we observed a significant improvement in the flatness value. The differences between the SE-AC and CT-AC methods were less than 3% for the count recovery and less than 2% for the flatness value.

In order to quantify the recovery of counts after AC on the myocardium and left ventricle, we acquired both mouse and rat images using ^18^F-FDG. Results showed that the SE-AC method leads to difference in count recovery of about 3% and 10% to the CT-AC method for mouse and rat, respectively. We evaluated also the recovery of counts in a mammary tumour in a mouse showing a difference of RV with respect to CT AC method of about 1.5%. The accuracy of the SE-AC method was also evaluated on rat images acquired with ^11^C-acetate, ^68^Ga-chloride, and ^18^F-NaF. The difference between RVCT and RVSE measured in liver on ^11^C-acetate image is approximately 11%. The ROI analysis performed on ^68^Ga-chloride image on the kidneys leads to a difference of about 7% between CT-AC and CT-AC. ROI analysis on spinal column of ^18^F-NaF images showed a difference in RV of about 20%. This slightly higher difference between CT-AC and SE-AC when using ^18^F-NaF images is due to more difficulties in contour delineation during the segmentation procedure and also to the error one makes when assigning to bone tissues as seen in ^18^F-NaF scans the attenuation coefficient of soft tissues. In vivo studies showed that the RV values obtained by using SE-AC are systematically lower than CT-AC ones, except in tumour mouse study where the RV values are quite similar for both AC methods.

## 5. Conclusions

The main goal of the paper was to show that SE-AC provides comparable results with respect to the more complex and time-consuming CT-AC method. We proved this for several radiotracers used in preclinical imaging such as ^18^F-FDG, ^11^C-acetate, ^68^Ga-chloride, and ^18^F-NaF. The SE-AC method has, however, some limitations and will probably fail when a very specific tracer is used. In this case, CT-AC method must be applied. 

As expected, AC is more important for rat than for mouse images. For all the studies performed on animals, we observe a difference of RV between CT-AC and SE-AC method of less than 10%, except for ^18^F-NaF images where the deviation is 20%. In all cases, RV estimated from SE-AC is smaller than the one estimated from CT-AC. Overall, we can conclude that the SE-AC method provides good quantitative estimates of count recovery, comparable to the ones obtained from CT-AC. This is important considering the advantages of the SE-AC: (1) it requires less time with respect to the standard method based on CT images, because the CT image acquisition and, consequently, the coregistration procedure are not needed; (2) it does not add dose to the animal, and this is very important when repeated studies are needed; (3) it can be used also when a small animal CT scanner is not available.

## Figures and Tables

**Table 1:** Density and attenuation coefficient used to calibrate the CT scanner. Data were calculated by Hubbell and Seltzer and were published by the National Institute of  Standards and Technology [26].

Material	Density (g · cm^−3^)	Attenuation coefficient at 511 keV (cm^−1^)
Air	0.001	0
Polyethylene	0.930	0.0925
Polystyrene	1.060	0.0994
Water	1.000	0.0969
Acrylic	1.190	0.1120
Polytetrafluoroethylene (Teflon)	2.250	0.1886
Aluminum	2.699	0.2280

**Table 2:** Average number of counts and recovery value measured on uniformly filled mouse and rat phantom images and in the hot sphere of a rat-sized phantom. For the uniformly filled phantoms, also the flatness is showed.

	Roi counts (cps)	RV (%)	Flatness
Mouse phantom			
Original PET image	429.68	—	3.1%
CT-AC image	623.02	31.0%	1.1%
SE-AC image	613.89	29.6%	1.2%
Rat phantom			
Original PET image	210.34	—	22.0%
CT-AC image	405.32	48.1%	1.2%
SE-AC image	396.17	45.8%	2.7%
Rat phantom with hot sphere			
Original PET image	452.79	—	—
CT-AC image	760.83	40.5%	—
SE-AC image	708.37	33.6%	—

**Table 3:** ROI counts measured in an ROI inside the tumour of a mouse obtained using ^18^F-FDG images (the uncorrected and the two AC images) and recovery value (see (4)) calculated with the two AC methods.

	Roi counts (cps)	RV (%)
Original PET image	527.82	—
CT-AC image	716.94	26.4%
SE-AC image	726.13	27.7%

**Table 4:** ROI counts measured on the myocardium and in the left ventricle obtained using ^18^F-FDG rat images. The RV calculated with (4) is shown for both CT-AC and SE-AC images.

	Roi counts (cps)	RV (%)
Original PET image		
Myocardium	3 463.70	—
Ventricle	1 975.00	—
CT-AC image		
Myocardium	4 478.60	22.7%
Ventricle	2 577.30	23.4%
SE-AC image		
Myocardium	4 393.40	20.8%
Ventricle	2 511.30	20.8%

**Table 5:** Counts measured in an ROI inside the left ventricle and inside the myocardium of the ^18^F-FDG rat image and of the AC ones. The recovery values calculated using (4) are shown in the last column of the table for both AC methods.

	Roi counts (cps)	RV (%)
Original PET image		
Myocardium	520.35	—
Ventricle	352.63	—
CT-AC image		
Myocardium	858.38	39.4%
Ventricle	589.99	40.2%
SE-AC image		
Myocardium	785.43	30.9%
Ventricle	537.87	31.4%

**Table 6:** Average values of coincidences calculated in an ROI inside the liver on ^11^C-acetate rat images. The recovery values estimated with the two AC methods are shown in the last column of the table.

	Roi counts (cps)	RV (%)
Original PET image	174.92	—
CT-AC image	327.04	46.5%
SE-AC image	291.56	35.7%

**Table 7:** Average values of counts measured in an ROI inside the kidney from a ^68^Ga-chloride rat PET image. The RV computed from both AC methods is shown in the last column of the table.

	Roi counts (cps)	RV (%)
Original PET image	245.45	—
CT-AC image	459.14	46.5%
SE-AC image	427.37	39.6%

**Table 8:** ROI average values computed from ^18^F-PET image of a rat corresponding to the spinal column for uncorrected and AC images. In the last column of the table, the RV from both AC methods is shown.

	Roi counts (cps)	RV (%)
Original PET image	419.70	—
CT-AC image	770.14	45.5%
SE-AC image	623.67	26.5%
